# Functional and therapeutic effects of Glabrescione B delivery by liposomes on Hedgehog-dependent tumors

**DOI:** 10.1007/s13346-025-02026-0

**Published:** 2025-12-16

**Authors:** Paola Infante, Raffaella Daniele, Marta Bottero, Mariagrazia Longo, Francesca Bufalieri, Ludovica Lospinoso Severini, Cristiano Pesce, Agnese Fragassi, Daniela Gabbia, Shirin Navacci, Irene Basili, Gennaro Adabbo, Silvia Cammarone, Gabriele Cianfoni, Francesca Ghirga, Mattia Mori, Sara De Martin, Mariateresa Mancuso, Paolo Caliceti, Simonetta Pazzaglia, Stefano Salmaso, Lucia Di Marcotullio

**Affiliations:** 1 Department of Molecular Medicine, Sapienza University, Rome, Italy; 2 Department of Pharmaceutical and Pharmacological Sciences, University of Padova, Padova, Italy; 3 Institut Curie, PSL Research University, INSERMU1330/CNRS EMR 8001. Children’s Oncology Research Unit (CONCERT), Paris, France; 4 CNRS EMR 8001. Children’s Oncology Research Unit (CONCERT), Université Paris Sud, Université Paris-Saclay, INSERMU1330 Paris, France; 5 Istituto Pasteur-Fondazione Cenci Bolognetti, Sapienza University of Rome, Rome, Italy; 6 Dipartimento Di Chimica E Tecnologie del Farmaco, Sapienza University, Rome, Italy; 7 Department of Biotechnology, Chemistry and Pharmacy, University of Siena, 53100 Siena, Italy; 8 Division of Biotechnologies, Italian National Agency for New Technologies, Energy and Sustainable Economic Development (ENEA), 00123 Rome, Italy

**Keywords:** Liposomal delivery, Medulloblastoma, Basal Cell Carcinoma, Hedgehog signaling, Gli1 inhibitors

## Abstract

Aberrant Hedgehog (HH) signaling pathway is responsible of tumorigenesis of medulloblastoma (MB) and basal cell carcinoma (BCC), two aggressive malignancies with limited therapeutic options. Targeting Gli1, the final and powerful effector of HH signaling, emerged as a valuable strategy for the treatment of HH-dependent tumors. Among Gli1 inhibitors, Glabrescione B (GlaB), is a small molecule that directly inhibits Gli1/DNA interaction, which showed promising pre-clinical results. However, poor solubility limits its clinical translation. To overcome this issue, here we develop a liposomal formulation of GlaB (Lipo/GlaB) with optimized composition to enhance drug loading, controlled release, storage stability and pharmacokinetic performance. Among various formulations, liposomes composed of EPC and cholesterol (95:5 mol/mol%) achieves high GlaB loading efficiency and stability upon lyophilization. Lipo/GlaB inhibits Gli1 transcriptional activity more potently than free GlaB and significantly reduces the expression of HH target genes. Notably, Lipo/GlaB remarkably reduces the tumor growth in HH-driven MB and BCC in in vitro and in vivo models, correlating with decreased HH signaling. Further, pharmacokinetic studies in mice revealed improved plasma disposition, higher AUC, and slower elimination for Lipo/GlaB compared to the free drug. These findings support the therapeutic value of Lipo/GlaB as a selective and potent strategy for targeting HH-dependent cancers, offering improved biopharmaceutical properties and in vivo efficacy compared to non-formulated GlaB.

**Graphical Abstract:**

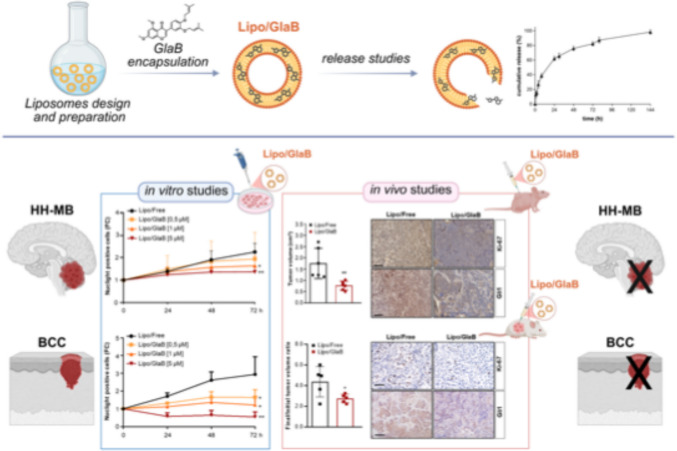

**Supplementary Information:**

The online version contains supplementary material available at 10.1007/s13346-025-02026-0.

## Introduction

Over the past years, Hedgehog (HH) signaling has moved to the forefront of molecular oncology, not because of its well-established developmental roles, but due to its recurrent and actionable dysregulation across multiple solid tumors. HH pathway activation sustains tumor cell proliferation, stemness, metabolic plasticity, and therapy resistance, making it an appealing point of therapeutic intervention [1–3]. Two tumor types exemplify the clinical relevance of HH hyperactivation: Medulloblastoma (MB) and Basal Cell Carcinoma (BCC). MB is a highly aggressive pediatric brain malignancy in which the HH molecular subgroup is defined by constitutive pathway activation, whereas BCC, the most common skin cancer, is driven in the majority of cases by genetic alterations leading to persistent HH signaling [4, 5]. In both settings, the tumor’s dependence on HH signaling creates a clear opportunity for targeted therapeutic strategies aimed at disrupting this oncogenic axis.

Current therapeutic approaches in HH-dependent MB involve surgery followed by radiation and/or chemotherapy, treatments frequently associated with significant toxicity and long-term neurological impairment [6]. Similarly, locally advanced BCCs that are not eligible for surgery or radiotherapy require chemotherapeutic treatments, while advanced or recurrent lesions still present significant clinical challenges [7]. In both malignancies, genetic or epigenetic alterations leading to constitutive HH signaling are primarily associated with their onset and tumorigenesis, thus making this pathway a compelling target for pharmacological intervention [8, 9].

Initial therapeutic efforts have focused on inhibiting HH signaling at the level of Smoothened (SMO), a key upstream signal transducer. However, the clinical efficacy of SMO inhibitors has been limited by the emergence of drug-resistant SMO mutations, activation of downstream signaling independent of SMO, as well as severe side effects [10]. Consequently, direct inhibition of the terminal transcriptional effector of the pathway, Gli1, has emerged as an attractive alternative strategy [11]. Glabrescione B (GlaB) is the first small molecule able to impair the HH oncogenic activity by directly inhibiting Gli1/DNA interaction, thus blocking the growth of Hedgehog-dependent MB both in vitro and in vivo [12, 13]. Nevertheless, GlaB suffers from poor water solubility, which affects its clinical use. Over the past decade, advances in drug delivery technologies have provided powerful tools to address these limitations, raising promises for translation of anticancer drugs to medicines by improving the drug biopharmaceutical features, without compromising the effect on molecular targets. Nanocarrier systems, such as liposomes, polymeric nanoparticles (NPs), bio-based NPs, and inorganic NPs, modulate drug stability, absorption, pharmacokinetic profile, biodistribution, and extend the exposure of tumors (controlled release) while minimizing that of healthy tissues (selective drug delivery) and facilitate the administration of synergistic drug combination [14].

To overcome the poor biopharmaceutical properties of GlaB, we took advantage of innovative nanopharmaceuticals and we developed a micellar formulation using the biocompatible compound methoxy-poly (ethylene glycol)-cholane (mPEG-Cholane), which increases the GlaB solubility by a 100-fold. This enables administration of the required dose in a small volume without the need for organic solvents [15, 16]. Importantly, the micellar formulation was found to increase the GlaB anticancer effects at lower doses compared to non-formulated GlaB, both in in vitro and in vivo models of HH-dependent MB [15]. The therapeutic relevance of these results prompted us to explore engineered liposomes to enhance the GlaB controlled delivery, further improving its biopharmaceutical features and the anticancer efficacy. Indeed, micelles may undergo dissociation upon parenteral administration which limits the control of the cargo delivery. Instead, the higher structural integrity of liposomes compared with PEG-lipid micelles in biological fluids [17–20] can, ultimately, extend the drug permanence in the bloodstream and its disposition in tumors. Liposome composition was systematically screened to select the colloidal formulation yielding the highest drug loading while retaining favorable biopharmaceutical features, including sustained payload release to achieve Gli1 transcriptional inhibition, as the consequence of the reduction of HH signature.

Our studies strongly support the capability of liposomal formulation of GlaB to suppress the Gli1 transcriptional activity and HH pathway, thus robustly suppressing the tumor growth in preclinical models of HH-driven MB and BCC.

## Materials and methods

### Materials

Hydrogenated soybean phosphatidylcholine (LIPOID S PC-3, HSPC) and egg phosphatidylcholine (EPC) were a kind gift by Lipoid GmbH (Ludwigshafen, Germany). Dioleoyl-sn-glycero-3-phosphocholine (DOPC) was purchased from Avanti® (Avanti Polar Lipids, Birmingham, AL-USA). Acetonitrile (ACN), chloroform (CHCl_3_), methanol (CH_3_OH), and trifluoroacetic acid (TFA) were provided by Honeywell (Honeywell International Inc., Charlotte, NC-USA). Cholesterol, ammonium thiocyanate (NH_4_SCN), calcium chloride (CaCl_2_), sucrose, trehalose, mannitol, ferric chloride hexahydrate (FeCl_3_·6H_2_O), Potassium chloride (KCl) was purchased from Sigma-Aldrich (Merck KGaA, Darmstadt, Germany). “Ultrapure” water (mQ grade, 0.06 Siemens cm^−1^) was generated with Millipore Milli-Q® purification system (Merck KGaA, Darmstadt, Germany). Eagle’s Minimum Essential Medium, Fetal Bovine Serum, Phosphate Buffered Saline without Ca^2+^/Mg^2+^ solution, thiazolyl blue tetrazolium bromide, glutamine, penicillin/streptomycin solution were obtained from Sigma-Aldrich (Merck KGaA, Darmstadt, Germany).

### Screening of liposome composition for GlaB loading

GlaB loaded liposomes were prepared according to the thin-film hydration process reported in the literature [21]. Three phospholipids were tested at increasing cholesterol/phospholipid molar ratios (from 5 to 50 mol/mol%). Briefly, stock solutions of HSPC, EPC, DOPC and cholesterol in ethanol were prepared at a 6.67 mg/mL concentration. These solutions were used to generate the lipid film in a 10 mL round bottom flask by mixing appropriate volumes containing a fixed amount of phospholipids (25.6 μmoles) and increasing amount of cholesterol (1.28–12.8 μmoles range). GlaB dissolved in ethanol (2 mg/mL) was added to the ethanolic lipid mixtures to result in 5 mol/mol % with respect to phospholipids. The organic solvent was removed under reduced pressure using a rotary evaporator and stored overnight under vacuum. The lipid film was hydrated with 1 mL of mQ water resulting in final total lipid concentrations ranging from 26.9 to 38.4 mM. Formulations were extruded 11 times using an Avanti mini extruder (Avanti Polar Lipids, Inc. Alabaster, AL, USA) (heated to 60 °C for HSPC containing liposomes, r.t for EPC and DOPC containing liposomes) and equipped with a polycarbonate membrane with 0.4 μm pore size followed by 11 extrusions with polycarbonate membranes with 0.2 μm pore size. This process also allowed to remove the non-loaded GlaB that is insoluble in water (solubility: 20 ng/mL) [16]. Phospholipids concentration in the liposome formulations was quantified by Stewart assay [22]. GlaB loading was assessed by RP-HPLC using a Jasco system (Easton, MD, USA) equipped with a Phenomenex Luna C18 reverse-phase column (5 μm, 100 Å, 250 × 4.6 mm). The column was eluted with mQ water (eluent A) and acetonitrile (eluent B) both added of 0.05% v/v trifluoroacetic acid in a gradient mode from 50 to 95% eluent B in 21 min at a flow rate of 1 mL/min. The UV–Vis detector UV-2075 Plus (Jasco system, Easton, MD, USA) was set at 257 nm. The GlaB concentration was derived from a calibration line generated using GlaB solutions in acetonitrile at increasing concentration in the 0.5–15 µg/mL range. The areas of eluted GlaB peaks were integrated using Borwin Chromatography 1.5 software (Jasco system; Easton, MD, USA).

The loading efficiency (LE%) and loading capacity (LC%) were calculated according to the following equations:

1 $$LE(\%)=\frac{{GlaB}_{recovered}(mol)}{{GlaB}_{fed}\left(mol\right)\bullet phospholipid\;recovery(fraction)}(\%)$$

2 $$LC\left(\frac{mol}{mol}\%\right)=\frac{{GlaB}_{recovered}(mol)}{phospholipid\;recovered(mol)}(\%)$$

Size and size distributions were analyzed by Dynamic Light Scattering (DLS) using a Malver Zetasizer Ultra upon dilution of liposomes with 10 mM phosphate, 150 mM NaCl, pH 7.4 at 0.1 mg/mL lipid concentration. Size was recorded as Intensity. GlaB-free liposomes were also generated with the same procedure.

### GlaB release study

Freshly prepared GlaB-loaded liposomes (20 mg/mL, 95:5 mol/mol% EPC/Cholesterol) were obtained by film hydration. The liposome concentration was derived from phospholipid concentration assessed by Stewart assay as reported above. Liposome were diluted to 5 mg/mL with 10 mM phosphate, 150 mM NaCl, pH 7.4. 1 mL samples were transferred into a 50 kDa MWCO Float-A-Lyzer® and dialyzed against 5 L of 10 mM phosphate, 150 mM NaCl, pH 7.4. The external medium was replaced twice a day with fresh buffer. At scheduled time points, 20 μL volumes were withdrawn from the dialysis bag and the GlaB concentration was assessed by RP-HPLC as reported above.

### Lyophilization study

GlaB-loaded liposomes (95:5 mol/mol% EPC/Cholesterol) underwent lyophilization study. Trehalose, mannitol and sucrose tested as lyoprotectant were added to freshly prepared liposome dispersions. 0.5 mL of 20 mg/mL GlaB loaded liposomes were added of 0.5 mL of aqueous solutions of either trehalose, or mannitol or sucrose to yield lyoprotectant concentration in the 0–7% w/V range. The samples were frozen in liquid N_2_ and lyophilized overnight. Lyophilized formulations were then redispersed in 0.5 mL of mQ water and underwent size and size distribution analysis by DLS upon dilution to 0.1 mg/mL with 10 mM phosphate, 150 mM, pH 7.4.

GlaB leakage from liposomes after lyophilization was assessed. GlaB-loaded liposomes (20 mg) lyophilized with 3 w/V % sucrose were redispersed in 0.5 mL of mQ water, diluted to 5 mg/mL in 10 mM phosphate, 150 mM NaCl, pH 7.4 and filtered through 0.22 μm cellulose acetate membrane to remove the aggregated liposomes and GlaB precipitates. GlaB concentration was assessed by HPLC as reported above and phospholipid concentration was quantified by Stewart assay [22] to derive loading capacity.

GlaB-loaded liposomes lyophilized with 3 w/V % sucrose were reconstituted to 20 mg/mL in mQ water and after dilution to 5 mg/mL with 10 mM phosphate, 150 mM NaCl, pH 7.4 underwent release study as reported above.

### Colloidal stability

*Lyophilized liposomes.* GlaB loaded lyophilized liposomes (95:5 mol/mol% EPC/Cholesterol) were stored at 4 °C up to 6 weeks and, at scheduled time points, samples were first reconstituted to 20 mg/mL in mQ water and then diluted with 10 mM phosphate buffer, 150 mM NaCl, pH 7.4 to 5 mg/mL and analyzed for size and size distribution by DLS as reported above immediately after the reconstitution.

*Freeze-and-thaw stability*. 0.5 mL of 20 mg/mL freshly prepared GlaB loaded liposomes (95:5 mol/mol% EPC/Cholesterol) were added of 0.5 mL of sucrose aqueous solution (60 mg/mL) to yield 3% w/V sucrose concentration. The mixture underwent freeze-and-thaw cycles in liquid nitrogen. Size of liposomes was assessed after each thaw step by DLS as reported above.

*Stability under physiological mimicking conditions*. Lyophilized GlaB-loaded liposomes (20 mg, 95:5 mol/mol% EPC/Cholesterol) with 3 w/V % sucrose were reconstituted in mQ water to 20 mg/mL and then diluted to 5 mg/mL lipid concentration with either 10 mM phosphate, 150 mM NaCl, pH 7.4 or 10 mM phosphate, 150 mM NaCl, pH 7.4 containing 5 V/V % of FBS. Samples were thermostated at 37 °C using a thermomixer (Thermomixer Comfort, Eppendorf, Hamburg, Germany). Size and PDI of liposomes were assessed at scheduled time points for 72 h.

### Cell cultures

Mouse Embryonic Fibroblasts (MEFs) from wild-type (WT) and *Ptc*^−/−^ mice were cultured in Dulbecco’s Modified Eagle’s Medium (DMEM, Sigma-Aldrich, St. Louis, MO, USA) supplemented with 10% fetal bovine serum (FBS, Merck, Darmstadt, Germany). Human Daoy cells were cultured in Minimum Essential Medium Eagle (MEM, Sigma-Aldrich) supplemented with 2 mM L-glutamine, 1 mM sodium pyruvate, 100 IU/mL penicillin, 100 μg/mL streptomycin, and 0.25 μg/mL of amphotericin B, 10% (v/v) heat-inactivated fetal bovine serum (FBS, Merck). ASZ001 BCC cells were maintained in 154CF medium (Gibco-BRL, Grand Island, NY, USA) plus 2% FBS chelated with Chelex 100 sodium form (Sigma Aldrich), calcium chloride 0.05 mM (Gibco-BRL). Primary HH-MB cells were freshly isolated from Math1-cre/*Ptc*^fl/fl^ mice tumors. Briefly, tumor was mechanically disrupted with fire-polished Pasteur pipettes in HBSS (Sigma-Aldrich) with 1% Penicillin–Streptomycin (Pen/Strep) and treated with DNase (10 μg/ml) for 20 min. Cells were centrifuged and resuspended in Neurobasal Media-A (Thermo Fisher Scientific, Waltham, MA, USA) with B27 supplement minus vitamin A (Thermo Fisher Scientific), 1% Pen/Strep and 1% L-Glutamine and cultured in Neurobasal Media-A (Thermo Fisher Scientific) with B27 supplement minus vitamin A (Thermo Fisher Scientific). All media contained 1% L-Glutamine and 1% Pen–Strep (Sigma-Aldrich). Mycoplasma contamination in cell cultures was routinely detected by using PCR detection kit (Applied Biological Materials, Richmond, BC, Canada).

### Cytotoxicity studies

Daoy cells were seeded (3 × 10^3^ cells/well) in a 96-well tissue culture plate in complete MEM and were allowed to adhere overnight. Afterwards, the medium was replaced with 200 μL/well of complete medium containing reconstituted GlaB-loaded liposomes (95:5 mol/mol% EPC/Cholesterol) from lyophilized or equivalent free GlaB at concentrations in the 1–600 μM range. Cells were incubated for 72 h and then the medium was discarded, cells were washed 3 times with PBS. A 200 μL volume of complete medium was added per well followed by 20 μL of a 5 mg/mL thiazolyl blue tetrazolium bromide (MTT) solution in PBS and cells were incubated at 37 °C for 3 h. Afterwards, the MTT-containing medium was removed, and 200 μL/well of DMSO was added to dissolve the formazan crystals formed by the live cells. The plates were gently shaken for 15 min, and the absorbance was measured at 570 nm using a INNO-M microplate spectrophotometer (Ltek, Sangdaewon-dong, Seongnam-si, Gyeonggi-do, Republick of Korea). The cell viability was derived with respect to the viability of untreated cells. Sucrose solutions in complete medium at concentrations equal to that of samples containing the GlaB-loaded liposomes, and GlaB-free liposomes at equivalent lipid concentration of the GlaB-loaded liposomes were also tested as controls.

### Luciferase reporter assay

Gli1-induced transcriptional activity was evaluated in wild-type MEFs transfected with the 12xGliBS-Luc reporter and pRL-TK Renilla plasmid (used for normalization), along with either an empty control vector or a Gli1 expression vector. Cells were treated for 24 h with increasing concentrations of the Lipo/GlaB (95:5 mol/mol% EPC/Cholesterol) formulations. Luciferase and Renilla activities were measured using a dual-luciferase assay system (Biotium Inc., Hayward, CA, USA), following the manufacturer’s instructions. Results are presented as luciferase/Renilla ratios and represent the mean ± S.D. of three independent experiments, each performed in triplicate.

### mRNA expression analysis

The mRNA expression levels of HH target genes were analyzed in validated HH-driven models characterized by constitutive pathway activation and sustained Gli1 expression. After the treatment period with Lipo/GlaB or Lipo/Free, as control, the total RNA was extracted using Trizol reagent (Thermo Fisher Scientific) and reverse-transcribed into cDNA using the SensiFAST cDNA Synthesis Kit (Bioline Reagents Limited, London, UK). Quantitative real-time PCR (qPCR) was performed on each cDNA sample using the ViiA™ 7 Real-Time PCR System (Life Technologies) to assess the expression of *Gli1*, *Gli2*, *Ptc*, *CycD1*, *CycD2*. Amplifications were carried out in triplicate using the SensiFAST Probe Lo-ROX Kit (Bioline) and FAST qPCR thermal cycling conditions. Data were analyzed with SDS software v2.3, and expression levels were calculated based on the average threshold cycle (Ct) values. Results were normalized to two endogenous controls, *β2-microglobulin* and *Hprt*, and expressed as relative mRNA levels (target gene/housekeeping gene ratios). For each target gene relative expression levels were calculated using the geometric mean of *β2-microglobulin* and *Hprt* Ct values. The sequences of the used primers are reported in Table S1.

### Immunoblot analysis

Protein lysates from HH-MB and HH-BCC tumor masses were prepared using RIPA buffer (50 mM Tris–HCl, pH 7.6; 150 mM NaCl; 0.5% sodium deoxycholate; 5 mM EDTA; 0.1% SDS; 100 mM NaF; 2 mM NaPPi; 1% NP-40) supplemented with protease and phosphatase inhibitors. The lysates were then centrifuged at 13,000 rpm for 30 min at 4 °C. Supernatants were collected and boiled in loading buffer for 5 min. Proteins were resolved by SDS-PAGE, transferred onto nitrocellulose membranes (GVS North America, Sanford, ME, USA), blocked with 5% skimmed milk in TBS containing 0.1% Tween 20 (Sigma-Aldrich), and probed with the specified antibodies. Actin and Hsp-70 have been used as loading controls. The details of the antibodies used are reported below: mouse anti-Gli1 (L42B10, 1:500) was purchased by Cell Signaling Technology (Beverly, MA, USA); β-Actin HRP (sc-47778, 1:1000) and mouse anti-HSP70/HSC70 (w27) (sc-24, 1:1000) were purchased by Santa Cruz Biotechnology (Santa Cruz, CA, USA). HRP-conjugated secondary antibodies were purchased by Bethyl Laboratories (Waltham, MA, USA).

### Cell proliferation assay

ASZ001 BCC cells (3 × 10^4^ cells/well) or primary murine HH-MB cells (2 × 10^4^ cells/well), were seeded onto a 96-well tissue culture plate in 100 μl complete medium (6 wells for each experimental point) and treated for 72 h with Lipo/GlaB or Lipo/Free (95:5 mol/mol% EPC/Cholesterol), as control, at the indicated concentrations. Cell proliferation was indicated as relative Nuclight staining (Nuclight Rapid Red reagent, #4717, Sartorius, Gottinga, Germany), calculated using the IncuCyte® Zoom software (Essen BioScience Ann Arbor, MI, USA). Cells proliferation is normalized to scans obtained at time 0 (T0) and expressed as fold change (FC) ± SD of *n* = 3 experiments.

### Animal studies

*Allograft experiments.* Spontaneous HH-MB derived from Math1-cre/*Ptc*^fl/fl^ mice, already available in our laboratory and generated by interbreeding Math1-cre and *Ptc*^fl/fl^ mouse lines, both originally purchased from The Jackson Laboratory (Maine, USA). HH-MB tumor mass was isolated, minced and pipetted to obtain a single-cell suspension. Equal volumes of cells (2 × 10^6^) were injected subcutaneously (s.c.) into the posterior flank of female BALB/c nude mice (nu/nu) (Charles River Laboratories, Lecco, Italy). When the tumor size reached a median size of ~ 150–200 mm^3^, the mice were randomly divided into two groups (*n* = 6/each group) and treated peritumorally every other day with Lipo/GlaB (9 mg/kg) or Lipo/Free (95:5 mol/mol% EPC/Cholesterol) as control for three weeks. Tumor growth was monitored by measuring the size by caliper and tumor volume was calculated by the formula length × width × 0.5 × (length + width).

*Animal irradiation. Ptc1*^±^ mice were irradiated at postnatal day 2 (P2) with a single dose of 10 Gy of x-rays. For irradiation, pups were marked on dorsal skin and individually placed inside a cylindrical lead shield designed to provide protection of the anterior two-thirds of the body. This was done to avoid induction of medulloblastoma, a radio-inducible tumor, with high penetrance and short latency that would have killed most of the mice at young age, thus preventing development of BCC, that is instead characterized by a longer tumor latency [23–25]. Irradiation was performed using a Gilardoni CHF 320 G x-ray generator (Gilardoni, Mandello del Lario, Lecco, Italy) operated at 250 kVp, 15 mA, with filters of 2.0 mm Al and 0.5 mm Cu (Half-Value Layer Z 1.6 mm Cu). Under these conditions, tumors typically begin to develop around 16 weeks of age, within a time window of approximately 8 months. Mouse skin was observed weekly for BCC development, which appeared as a small, raised papule or nodule on the skin surface, flesh-colored to pinkish. The inner surface of the excised skin revealed tumor infiltration into the dermis and underlying tissue. Treatment was initiated once tumors became palpable corresponding to a tumor volume of about 100–200 mm^3^. BCC tumors (*n* = 5/each group) were treated via intratumoral injection with either Lipo/GlaB (9 mg/kg) or Lipo/Free (95:5 mol/mol% EPC/Cholesterol) formulations, administered three times per week for five weeks. Tumor volume was assessed by measuring with a caliper the length and width of the tumor (Vt = 0,5 × L x W2) twice a week. After the treatment period, mice were euthanized. Tumor tissues were either snap-frozen for molecular analyses or collected and processed for histological examination.

*Pharmacokinetcs study of GlaB loaded liposomes.* The PK study was performed as reported in the literature [15]. C57BL/6 J mice (10 ± 1 week old) were housed in a temperature and humidity-controlled room under a constant 12 h light/dark cycle, with free access to water and food. The animals were randomly assigned to two experimental groups (*n* = 4) and 2.5 mg/kg of 0.41 mg/mL GlaB in 3.3% v/v of DMSO, 3% v/v of Cremophor, 4% v/v of Tween® 80, 15% v/v of PEG400, 20 mM phosphate buffer, 0.15 M NaCl, pH 7.4 or GlaB equivalent dose of lyophilized GlaB-loaded liposomes (95:5 mol/mol% EPC/Cholesterol) resuspended in 0.5% w/V sterile NaCl solution, were intravenously injected into the tail vein. Blood samples (0.1 mL) were collected from the submandibular plexus at regular intervals using heparinized tubes: 5, 30 min, 1, 2, 4, 6, 8, 24 and 48 h after administration. During blood sampling, mice were kept under isoflurane anesthesia. The blood samples were centrifuged for 3 min at 2,000 rpm and GlaB in plasma was assessed using a 3000 UltiMate HPLC (Thermo Fisher Scientific, Whaltam, MA, USA) coupled to an AP 4000 Applied Biosystems LC–MS system (Sciex, Framingham, MA, US). Ten μL of plasma were added to 290 μL of acetonitrile supplemented with 0.5 μg/mL of benzanilide (internal standard) and centrifuged at 3,700 rpm for 15 min. The supernatant was analysed by using a reversed-phase column Phenomenex Gemini C18 (5 μm, 110 Å 50 × 2 mm) eluted at a flow of 0.3 mL/min with ammonium formate buffer (10 mM, pH 3.5, eluent A) and acetonitrile (eluent B) with the following gradient: from 60 to 95% of eluent B in 1 min. The GlaB concentration was derived from a calibration line obtained using 60–36,000 pg/mL GlaB solutions in plasma. GlaB plasma concentration vs time data were analysed using GraphPad Prism 10.02 software (San Diego, CA, USA) according to the Akaike Information Criterion. A bi-exponential equation was the best-fitting model for all the pharmacokinetic data obtained in this study. Pharmacokinetic parameters were calculated from the coefficients and exponents of the best-fits by using standard formulae. All animal experiments were approved by local ethics authorities (Ministry of Health) and conducted in accordance with Italian Governing Law (D.lgs 26/2014).

### Immunohistochemistry

For immunohistochemistry sections (4 µm) of paraffin-embedded BCC tumors and MB allograft tumors, the tissues were prepared following the standard protocol. Briefly, sections were dewaxed for 13 min at 90 °C with Heat Mediated Antigen Retrieval Solution (“HMARS”, Abcam, Germania) pH 6.0. Afterward, sections were washed in water for 5 min and peroxidases inhibited by incubation in 3% H_2_O_2_ for 10 min. Sections were treated with 5% bovine serum albumin (Santa Cruz Biotechnology, Santa Cruz, CA) diluted in phosphate-buffered saline (PBS) for 30 min and incubated with the primary antibodies: anti-Ki67 (IHC-00375, Bethyl, USA, 1:200). Sections were then washed in PBS, incubated for 1 h at room temperature with the secondary anti-rabbit antibody (Dako North America, Carpinteria, CA) diluted 1:200 in PBS, and washed again. To visualize the antigen, sections were incubated with Vectastein Elite ABC (Vector Laboratories, Inc., Burlingame, CA) for 30 min at room temperature, washed with PBS and incubated with Vector NovaRED Substrate Kit (Vector Laboratories, Inc., Burlingame, CA) for 15 min at room temperature. Finally, samples were stained with H&E and analyzed by light microscopy.

### Statistical Analysis

The statistical analysis of data was carried out by Graph Pad Prism 9 software. Statistical analysis of tumor volume was performed using an unpaired t-test. Mean ± standard deviation (SD) of the results was based on at least three experiments. Other analyses were performed by using Two-way ANOVA or One-way ANOVA, when appropriate, followed by Dunnett’s post hoc test when comparing multiple treatments to a single control, or Tukey’s post hoc test when comparing all groups to each other. A p value < 0.05 was considered statistically significant.

## Results and discussion

### Selection of the liposome composition

**Fig. 1:**
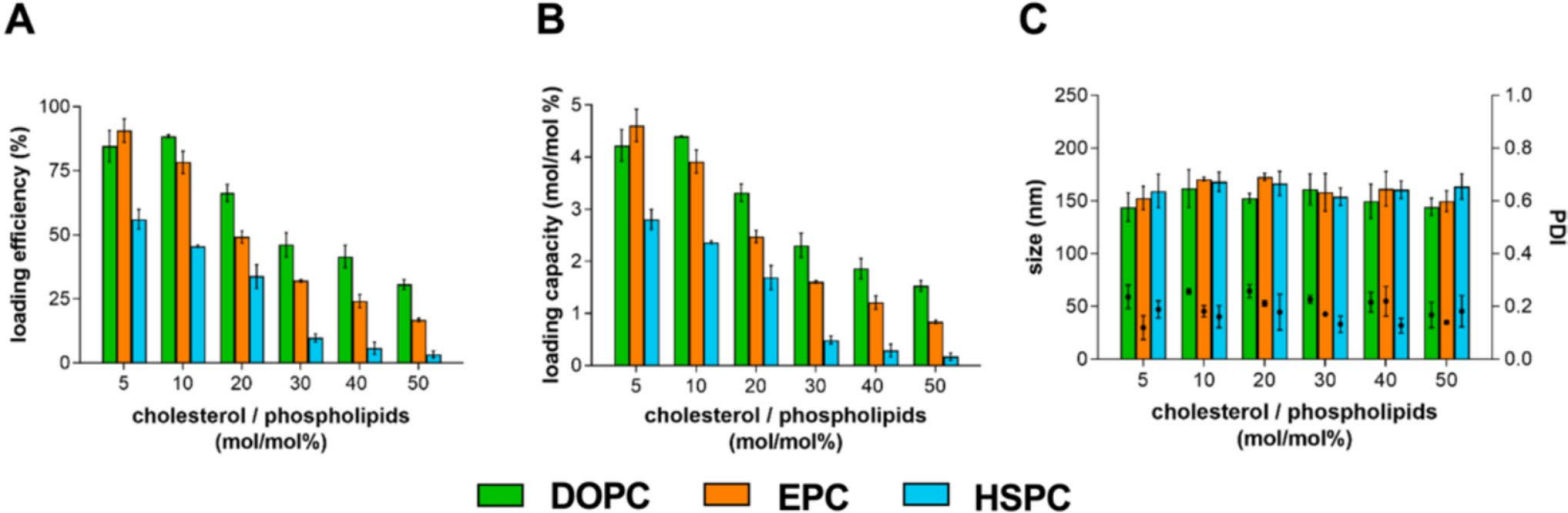
Selection of the liposome composition. GlaB loaded liposomes obtained with different phospholipids at increasing cholesterol/phospholipids molar ratios: (**A**) loading efficiency; (**B**) loading capacity; (**C**) size (columns) and PDI (•). Data are reported as means ± standard deviation calculated with four test repetitions

Liposomes developed in this study have been designed to yield a GlaB formulation with enhanced biopharmaceutical features compared to micelle formulations previously developed by using mPEG-Cholane [15]. The formulation composition was investigated to achieve colloidally stable products and controlled delivery of GlaB to Hedgehog-dependent tumors. GlaB is a small polycyclic hydrophobic molecule with high LogP (6.1 computed value by XLogP3 3.0 software, PubChem release 2019.06.18), which makes it a good candidate for liposome encapsulation through lipid bilayer intercalation [26]. Previous studies highlighted that unsaturation degree of phospholipids and cholesterol/phospholipid ratio dictate the loading of hydrophobic drugs into the liposomal bilayer [27–32]. Inspired by these evidences, we first investigated the loading of GlaB into liposomes obtained using three phospholipids with different degree of unsaturation: 1. DOPC, a phosphatidylcholine with 18 carbons in the fatty acid chains with one unsaturation per chain; 2. EPC as representative of phosphatidylcholine with prevailing 18 carbon atoms fatty acids (~ 60%) with different degrees of unsaturation (26.2% saturated, 48.3% mono-unsaturated, and 25.5% with two unsaturation sites) [33]; 3. HSPC as representative of fully saturated semisynthetic phosphatidylcholine with prevailing 18 atoms of carbon chains (~ 90% 18 C) [34]. Libraries of GlaB loaded liposomes were prepared by using different phospholipid/cholesterol ratios using fixed amount of each of the three phospholipids while increasing cholesterol. GlaB loading was determined by HPLC using the calibration curve y = 132268x + 1707.2 (R^2^ = 0.9999); the analyte eluted at 17 min. According to preliminary studies showing poor loading efficiency at GlaB/phospholipids feed ratio above 5 mol/mol%, liposomes were prepared by using 5 mol/mol% GlaB/phospholipids feed ratio. Figure 1 shows the loading efficiency and capacity of the liposome libraries.

Cholesterol/phospholipid molar ratios lower than 5 mol% in the liposome composition were not tested in this screening, as HSPC-containing liposomes could not be processed at lower cholesterol ratios under the operative conditions used. The unsaturated phospholipids (i.e. DOPC and EPC) significantly enhanced the GlaB loading in liposomes with respect to the saturated HSPC. These results are in agreement with studies reported in the literature for hydrophobic drug, such as paclitaxel, of which loading in liposomes was higher when unsaturated phospholipids were used with respect to saturated counterparts [35]. Accordingly, liposomal paclitaxel formulations tested in the clinical trials (LEP-ETU® and EndoTag-1®) [36] and the marketed one Lipusu® are produced with unsaturated phospholipids (1,2-dioleoyl-sn-glycero-3-phosphocholine or EPC). Figures 1A and B show that the increase of cholesterol/phospholipids molar ratios in liposome composition remarkably decreased GlaB loading capacity and efficiency regardless the phospholipid saturation. This result is consistent with published studies showing that cholesterol negatively affects the loading of hydrophobic drugs (such as paclitaxel and celecoxib) into liposomes, as it competes with the drug for the hydrophobic domains of the liposome bilayers [37–39]. While the lipid composition provided different drug loading, no evident correlation was observed between the GlaB loading and the colloidal features of liposomes (Fig. 1C). All the compositions showed similar size of about 150 nm and size homogeneity with PDI lower than 0.25. In conclusion, the best formulation was obtained with 95:5 EPC/cholesterol molar ratio, which yielded 90.7 ± 4.5% GlaB loading efficiency and 4.6 ± 0.3% loading capacity, size of 152.8 ± 11.0 nm and PDI of 0.012 ± 0.04. This formulation was selected for further studies.

### GlaB release, lyophilization and colloidal stability

**Fig. 2:**
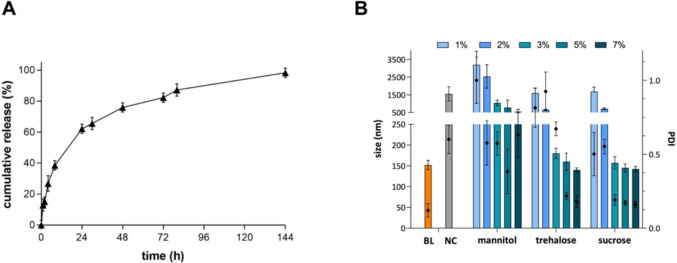
GlaB release, lyophilization and colloidal stability. (**A**) GlaB cumulative release from 95:5 EPC/cholesterol molar ratio GlaB-loaded liposomes in 10 mM phosphate, 150 mM NaCl, pH 7.4 at 37 °C. Mean ± Standard Deviation (SD) of the results were based on four test repetitions. (**B**) Size of 95:5 mol/mol% EPC/cholesterol GlaB-loaded liposomes redispersed after lyophilization. Lyophilization was performed in the presence of increasing lyoprotectant concentration (w/V%). The size of freshly prepared liposomes (BL) and of liposomes lyophilized without lyoprotectant (NC) are reported as references. PDI is displayed on the secondary right axis as black dots (●). Data are reported as means ± standard deviations based on four test repetitions. Statistical analysis is reported in Table S2

GlaB release was assessed under physiologically mimicking environment. Figure 2A shows that GlaB is slowly released from liposomes according to a diffusive process without burst release. About 60% of GlaB is released in 24 h and the complete release was achieved in 6 days.

Liposome lyophilization was explored to provide prolonged shelf-life of the formulation. Indeed, drug leakage can compromise the long-term storage stability of liposomes [35], while unsaturated lipids can undergo oxidation and degradation [40, 41]. Lyophilization is commonly employed to stabilize pharmaceutical formulations prone to physicochemical instability, including liposomal formulations such as AmBisome®. To identify the most effective conditions for preserving the colloidal and biopharmaceutical properties of GlaB-loaded liposomes, lyophilization was performed using three different lyoprotectants at varying concentrations. Figure 2B shows the results obtained both in the absence of lyoprotectants and in the presence of mannitol, trehalose and sucrose at increasing concentrations. Without lyoprotectants or at low concentrations (< 3%), liposomes undergo strong aggregation. Under these conditions, the size of the redispersed liposomes was > 500 nm and PDI > 0.5. In all cases, the liposome aggregation decreased with increasing lyoprotectant concentration. However, with mannitol, the size and PDI of the redispersed liposomes remained above 500 nm and 0.5, respectively, even when high sugar concentrations were used. Among the three lyoprotectants, sucrose demonstrated the best performance. The size of redispersed liposomes obtained by lyophilization with sucrose concentrations ≥ 3% w/V was comparable to the freshly prepared liposomes, while the PDI slightly increased, remaining below 0.2, a threshold generally considered acceptable for pharmaceutical liposome candidates [42].

Importantly, no GlaB leakage was detected upon redispersion of lyophilized liposomes, and the release profile closely matched that of fresh liposomes (Figure S1), indicating that freeze-drying does not induce alteration of the biopharmaceutical features of the formulation.

Based on these results, 3% w/V sucrose was selected as the lyoprotectant for further studies.

After lyophilization with 3% w/V sucrose, Lipo/GlaB redispersion in aqueous medium yielded up to 2.5 mM GlaB concentration, which is about 2 folds higher than the maximal concentration obtained with mPEG_5kDa_-cholane [15]. The redispersed liposomes maintained the same colloidal properties, mean size and PDI, and release profile of the liposomes before lyophilization.

The long-term stability of GlaB-loaded liposomes lyophilized with 3% w/V sucrose was evaluated over six weeks at 4 °C to simulate extended storage conditions (Figure S2A). The size of the reconstituted liposomes remained comparable to that of freshly prepared formulations over the range time of the study. Freeze-and-thaw studies showed that sucrose has also a cryoprotectant effect on aqueous liposomal dispersions. Without sucrose, the freeze-and-thaw procedure induces a strong liposome aggregation with particles and PDI increase with the freeze-and-thaw cycles. On the contrary, liposome dispersions containing 3% w/V sucrose maintain size and PDI of freshly prepared liposomes over six cycles (Figure S2B), confirming the cryoprotective effect of sucrose.

Taken together, these findings show that 3% w/v sucrose has both lyoprotectant and a cryoprotectant properties for GlaB-loaded liposomes, allowing the nanocarrier to be stored either as a lyophilized powder or as a frozen aqueous dispersion.

Before in vitro and in vivo investigations, the stability of GlaB-loaded liposomes was further assessed under physiologically mimicking conditions. The formulations exhibited excellent colloidal stability in physiological buffer either without or supplemented with fetal bovine serum (FBS) (Figure S2C). No alteration of particle size was observed over a 72-h incubation, indicating that the presence of salts and serum proteins did not induce liposomal aggregation.

### Cytotoxicity studies

The in vitro toxicity of GlaB loaded liposomes was tested on human MB cells [43]. Figure S3 shows the concentration-dependent sensitivity of human MB cells when treated with the liposomal GlaB with respect to the free drug.

Due to solubility limits, the free GlaB could only be tested up to100 µM in complete medium. The cytotoxicity profile of the GlaB loaded liposomes was slightly lower than that of the free drug, which is in agreement with the release profile reported above. Indeed, about 80% of the drug is released from the liposomal formulation in 72 h. GlaB loaded liposomes and free GlaB showed IC_50_ of 85.5 µM and 56.7 µM, respectively. GlaB-free liposomes and sucrose at equivalent concentration did not elicit toxicity within the concentration range used.

### Effect of Lipo/GlaB on Gli1 transcriptional activity and HH signature

**Fig. 3:**
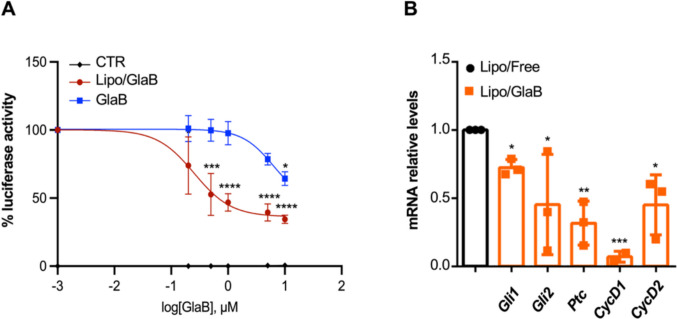
Effect of Lipo/GlaB on Gli1 transcriptional activity and HH signature. (**A**) Inhibition of Gli1-induced transcription was evaluated in WT MEFs transfected with 12XGli1-BS-Luc and pRL-TK Renilla (normalization control), along with the empty vector control or Gli1 vector. Cells were treated with increasing concentrations of Lipo/Free (CTR), GlaB dissolved in DMSO or Lipo/GlaB (0.2, 0.5, 1, 5, and 10 µM) for 24 h. Data are presented as the mean ± S.D. from three independent experiments. *p < 0.05; ***p < 0.001; ****p < 0.0001 vs CTR (Lipo/Free). (**B**) *Ptc*^−/−^ MEF cells were treated with Lipo/GlaB at the final concentration of 1 µM or Lipo/Free as control, and mRNA expression levels of the indicated genes were quantified by qRT-PCR and normalized to endogenous reference genes (*Hprt* and *β2-microglobulin*). *p < 0.05; **p < 0.01; ***p < 0.001 vs Lipo/Free

To evaluate the effect of GlaB formulated in liposomes (Lipo/GlaB) on Gli1 transcriptional activity we performed a luciferase reporter assay. To this end, Mouse Embryonic Fibroblasts (MEFs) were transiently transfected with ectopic Gli1 and a Gli-dependent luciferase reporter and then treated with increasing amounts of Lipo/GlaB or GlaB-free liposomes (Lipo/Free) as control. As shown in Fig. 3A, Lipo/GlaB treatment induced a dose-dependent reduction of Gli1 transcriptional activity, showing an IC_50_ of 1.081 μM, a value significantly lower than free GlaB-free dissolved in DMSO (IC_50_ 12 μM), as also previously reported [12].

Importantly, neither the GlaB-free liposomes nor the Lipo/GlaB formulations affected Renilla activity, which was used as an internal control, highlighting the non-toxic nature of both formulations to cells. Furthermore, to assess the impact of Lipo/GlaB on HH signature, we took advantage of MEF knockout for Ptc (*Ptc*^−/−^ MEFs), in which the loss of the repressive receptor Ptc1 determines the constitutive activation of HH signaling. The treatment of *Ptc*^−/−^ MEFs with Lipo/GlaB significantly downregulated the expression levels of the HH target genes *Gli*, *Gli2*, *Ptc*, *CycD1*, and *CycD2*, thus demonstrating the capability of this formulation to abrogate the activation of HH signaling (Fig. 3B).

### Lipo/GlaB inhibits the in vitro proliferation of HH-dependent MB and BCC models

**Fig. 4:**
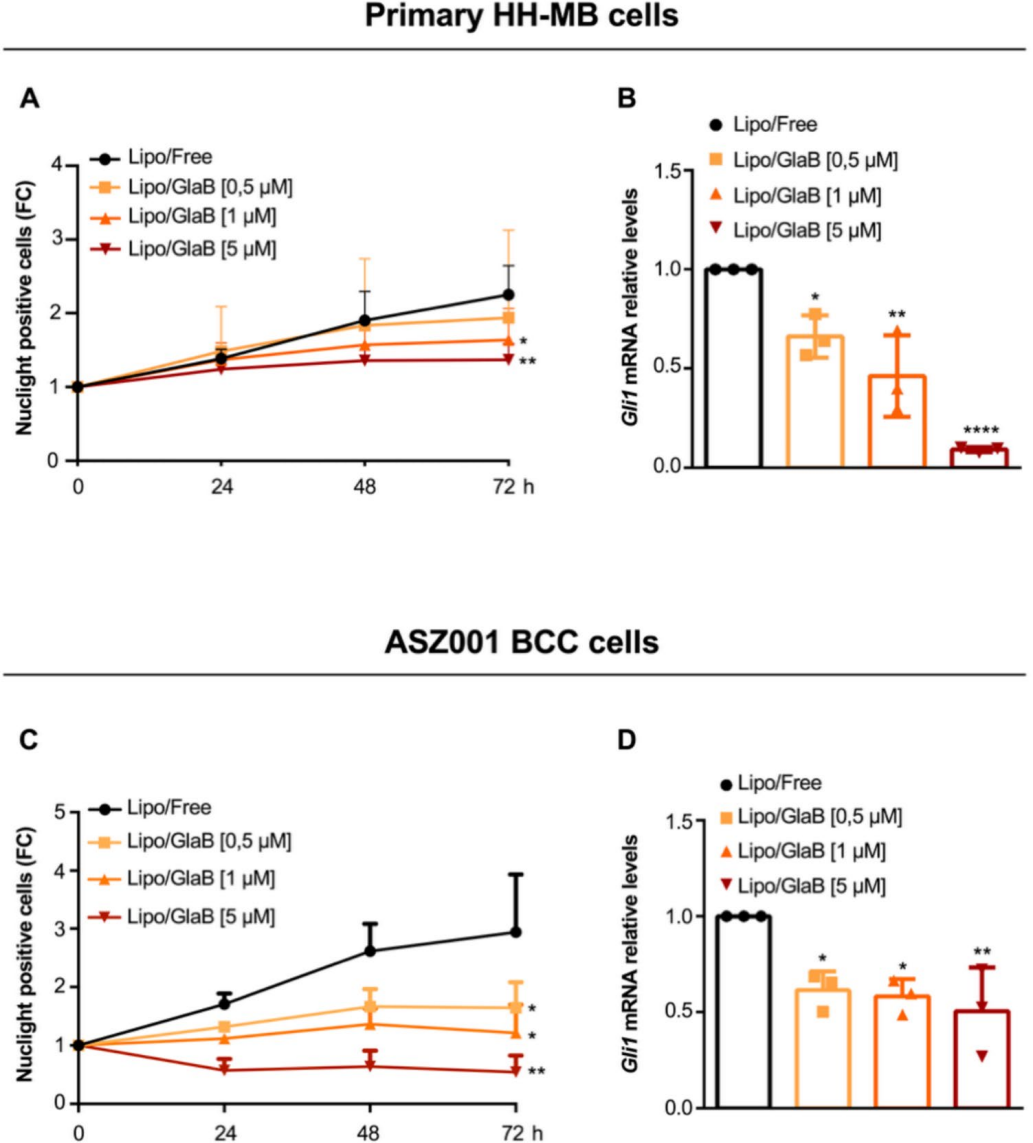
Lipo/GlaB inhibits the proliferation in vitro of HH-dependent MB and BCC models. (**A** and **C**) Primary HH-MB cells and ASZ001 BCC cells were treated with Lipo/Free or increasing concentrations of Lipo/GlaB (0.5 µM – 1 µM – 5 µM). Cell proliferation has been measured as relative Nuclight staining calculated using IncuCyte® Zoom software at the indicated time points. *p < 0.05; **p < 0.01 vs Lipo/Free. (**B** and **D**) *Gli1* mRNA expression levels were evaluated by qRT-PCR normalized to endogenous reference genes (*Hprt* and *β2-microglobulin*). *p < 0.05; **p < 0.01; ****p < 0.0001 vs Lipo/Free. Error bars indicate ± S.D

Aberrant activation of the HH pathway is primarily related to the tumorigenesis of medulloblastoma (MB) and Basal Cell Carcinoma (BCC). To demonstrate the ability of GlaB encapsulated in liposomes to suppress the proliferation of HH-dependent tumors, we firstly tested this formulation in in vitro well-established HH-driven MB and BCC cell models that exhibit constitutive HH pathway activation and Gli1 upregulation and are widely recognized in the literature as reliable models to evaluate Hedgehog/Gli1 inhibition [12, 15, 44]. In particular, we analyzed the anti-proliferative properties of Lipo/GlaB on primary HH-dependent MB cells freshly isolated from Math1-cre/*Ptc*^fl/fl^ mice that spontaneously developed MB and tested in short-term cultures to keep HH sensitivity in vitro [15]. To this aim, primary murine HH-MB cells were treated with increasing concentrations (0.5–1–5 µM) of Lipo/GlaB or GlaB-free liposomes (Lipo/Free) as control, and then the proliferation rate was assessed at different time points (24–48–72 h). As shown in Fig. 4A, Lipo/GlaB decreased the proliferation of primary HH-MB cells, as a consequence of a dose-dependent decrease in Gli1 expression levels (Fig. 4B).

Similar results were obtained also in ASZ001 BCC cells, previously characterized as an HH-dependent tumor cell line derived from *Ptc1* deletion [12, 44]. Notably, Lipo/GlaB induced a strong, dose-dependent reduction in HH-BCC cell growth compared to Lipo/Free (Fig. 4C), which correlated with a marked decrease in *Gli1* mRNA expression levels (Fig. 4D). Overall, these findings demonstrate that Lipo/GlaB exerts a potent in vitro anti-proliferative effect on HH-dependent tumor cells by inhibiting Gli1 expression, the most powerful final effector of the HH pathway.

### Lipo/GlaB suppresses the tumor growth in vivo of HH-driven MB and BCC mouse models

**Fig. 5:**
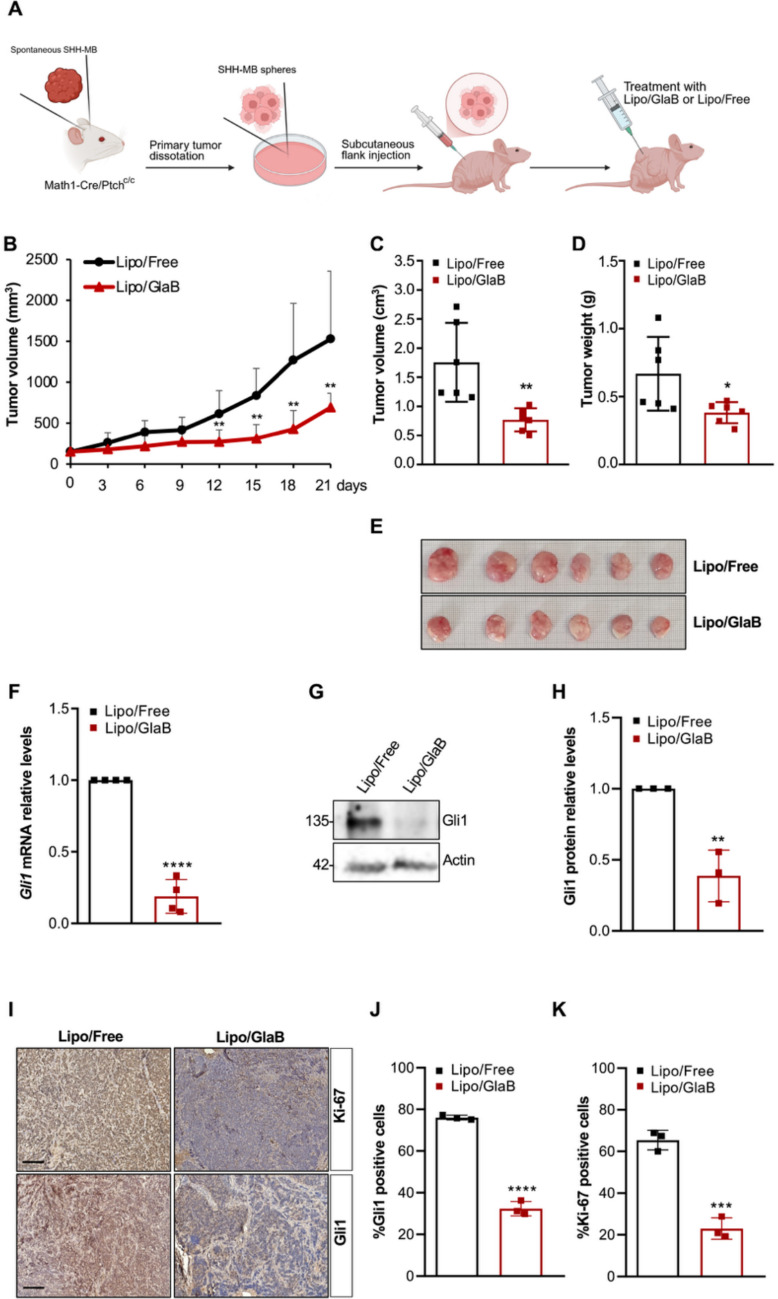
Lipo/GlaB reduces tumor growth of HH-dependent MB in in vivo allograft model. (**A**) Representative image of the procedure used to generate HH-MB allograft mouse model (created by using www.biorender.com). (**B**) Change of tumor volume during the period of treatment. Nu/Nu mice (*n* = 6 for each group) were grafted with spontaneous primary MB from Math1-cre/*Ptc*^fl/fl^ mice. Tumor masses were treated with Lipo/GlaB (9 mg/kg) or Lipo/Free as control. Tumor growth was monitored by caliper every third day. **p < 0.01 vs Lipo/Free. (**C** and **D**) Quantification of tumor volumes of explants (C) and weights (D). *p < 0.05, **p < 0.01 vs Lipo/Free. (**E**) Tumor masses after explantation. (**F**–**H**) qRT–PCR and western blot analysis showing the mRNA (F) and protein (G) expression levels of Gli1, respectively. For qRT–PCR, results were normalized to the endogenous controls (*Hprt* and *β2 microglobulin*). ****p < 0.0001 vs Lipo/Free. In (G) Actin was used as loading control. (H) Densitometric analysis of actin-normalized Gli1 protein levels shown in (G) of three independent experiments. **p < 0.01 vs Lipo/Free. (**I**) Immunohistochemical staining of Ki-67 (upper panel) and Gli1 (lower panel). Scale bar: 100 µm. (**J** and **K)** Quantification of Gli1 (J) and Ki-67 (K) stainings from immunohistochemistry shown in (I). *** *p* < 0.001, **** *p* < 0.0001 vs Lipo/Free. Error bars indicate ± S.D

To extend the evaluation of the therapeutic efficacy of Lipo/GlaB, we tested its anti-tumor properties using two in vivo models of HH-dependent tumors, MB and BCC, respectively. To establish the anti-cancer potential of Lipo/GlaB in an allograft model of HH-MB, we grafted nude mice posterior flank with primary HH-MB cells freshly isolated from Math1-cre/*Ptc*^fl/fl^ mice, and when the tumor masses were palpable, the mice were treated peritumorally with 9 mg/kg Lipo/GlaB or Lipo/Free (Fig. 5A). A progressive and marked reduction in tumor growth rate was observed over time (Fig. 5B) as well as in terms of tumor volume and weight (Figs. 5C-E) (at the end point of experiment) in the Lipo/GlaB-treated MB compared to the control. Accordingly, mice treated with Lipo/GlaB exhibited a consistent decrease in Gli1 mRNA and protein expression levels (Figs. 5F-J), as well as a significantly lower percentage of Ki67-positive proliferating cells, compared to those treated with Lipo/Free (Figs. 5I and K).

**Fig. 6:**
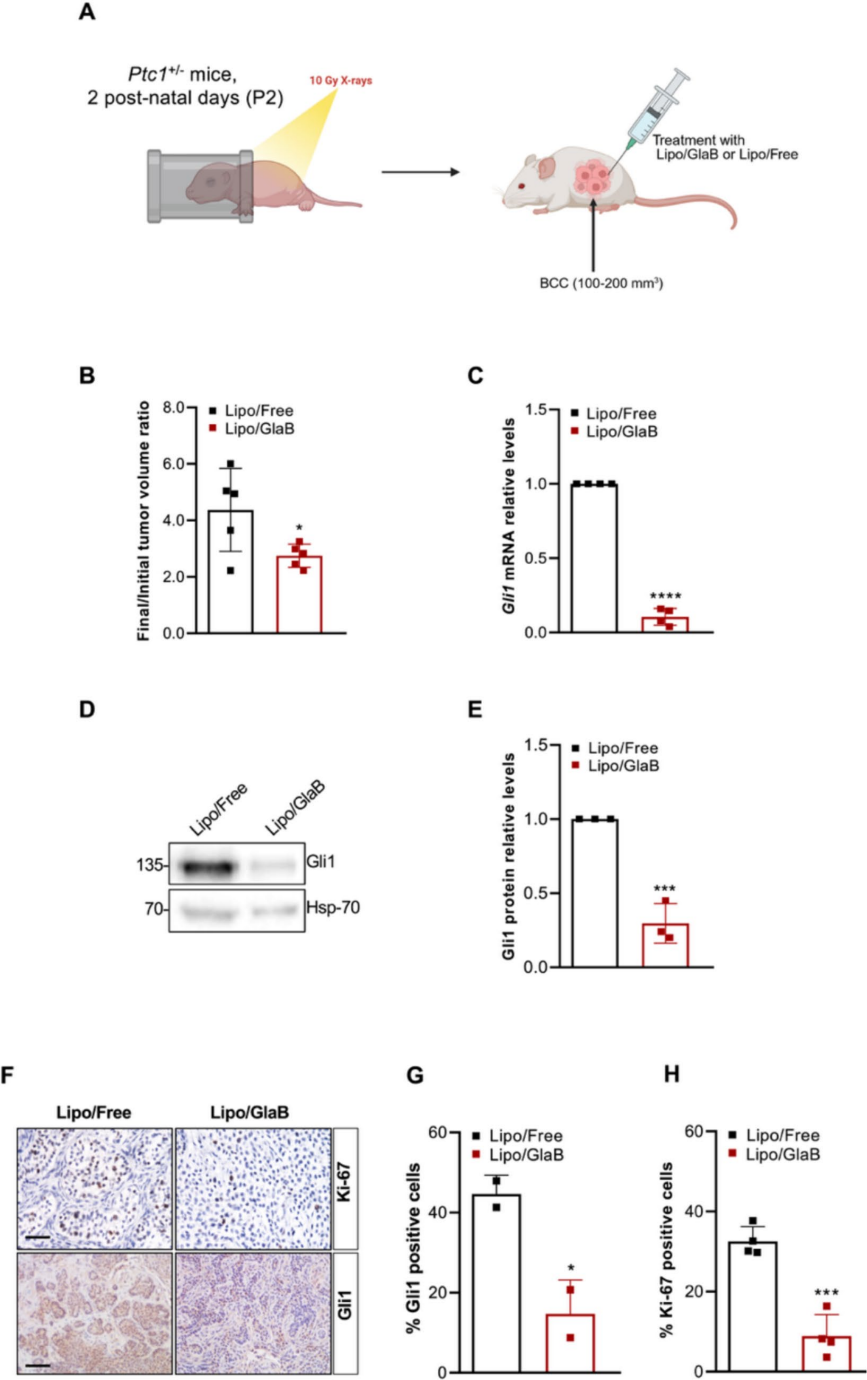
Lipo/GlaB suppress tumor growth of HH-driven BCC in vivo. (**A**) Representative image of the procedure used to generate HH-BCC mouse model (created by using www.biorender.com). *Ptc1*⁺/⁻ mice were exposed to a single 10 Gy dose of X-rays on postnatal day 2 (P2), using a cylindrical lead shield to protect the anterior two-thirds of the body, thereby specifically inducing BCC. (**B**) Ratio of final/initial tumor volume after treatment with Lipo/GlaB (9 mg/kg) and Lipo/Free in *Ptc1*^±^ mice irradiated on postnatal day 2 to induce BCC formation. *p < 0.05 vs Lipo/Free. (*n* = 5 for each group) (**C**-**E**) qRT–PCR and western blot analysis show the mRNA (C) and protein (D) expression levels of Gli1, respecitvely. For qRT–PCR, results were normalized to housekeeping genes (*Hprt* and *β2 microglobulin*). ****p < 0.0001 vs Lipo/Free. In (D) Hsp-70 was used as loading control. Densitometric analysis of Hsp70-normalized Gli1 protein levels shown in (D) of three independent experiments. ***p < 0.001 vs Lipo/Free (E). (**F**–**H**) Immunohistochemical staining of Ki-67 and Gli1 of tumor samples (F) and quantification of Gli1 (G) and Ki-67 (H) stainings from immunohistochemistry shown in (F). Scale bar: 100 µm. *p < 0.05; ***p < 0.001 vs Lipo/Free. Error bars indicate ± S.D

These data strongly support that Lipo/GlaB blocks the HH-driven MB growth in vivo, through the reduction of the HH pathway activity and consequently of tumor cell proliferation. Notably, when considering our previously reported in vivo data using free GlaB at an equivalent dose in the same MB model [11], the therapeutic effect observed with Lipo/GlaB is more pronounced. Indeed, at the endpoint the liposome formulation, admistered at 9 mg/Kg GlaB equivalent dose, yields a tumor reduction of about 50% higher with respect to free GlaB, thus supporting that liposomal encapsulation improves the in vivo performance of GlaB. In addition, Lipo/GlaB produced an antitumor response comparable to that previously obtained with the mPEG_5kDa_-cholane/GlaB micellar formulation, confirming that both systems are highly effective in delivering pharmacologically active GlaB to the tumor in vivo. To further validate these findings in an alternative in vivo context of HH-driven tumorigenesis, we utilized a *Ptc1*⁺/⁻ mouse model, which develop HH-BCC following a single 10 Gy dose of X-ray irradiation administered at postnatal day 2 (P2) and when the tumor masses became palpable, the mice were treated with Lipo/GlaB (9 mg/kg) or Lipo/Free intratumorally (Fig. 6A). This route of administration, as well as peritumoral injections performed for HH-MB allografts, ensures maximal tumor retention and allows assessment of the intrinsic therapeutic potential of the liposomal formulation in reachable tumor without the rapid off target biodistribution and clearance by organs including liver, spleen, and kidneys—phenomena that typically occur following intravenous (IV) administration. Our data, clearly demonstrated that Lipo/GlaB significantly reduced tumor progression compared to the control formulation (Lipo/Free), leading to an approximately 30% decrease in tumor growth rate (final-to-initial volume ratio) (Fig. 6B). Interestingly, this result correlated with the remarkable reduction in Lipo/GlaB treated compared to Lipo/Free BCC of Gli1 both mRNA (Fig. 6C) and protein expression (Fig. 6D and 6E), known readout of the HH pathway activity. Moreover, these findings are supported by immunohistochemistry for Gli1 and for the proliferative marker Ki-67, which revealed a significantly lower number of positive cells in Lipo/GlaB treated BCCs compared to controls (Figs. 6F-H). Importantly, this study represents the first evaluation of GlaB in a physiologically relevant de novo BCC model rather than in an allograft-based system, thereby strengthening the translational relevance of our findings and further supporting the therapeutic utility of liposome-encapsulated GlaB in the context of HH-dependent tumorigenesis. Overall, our in vivo evidence robustly supports the tumor-suppressive potential of GlaB formulated in liposomes for the treatment of HH-driven malignancies, such as MB and BCC.

### Pharmacokinetic study of GlaB-loaded liposomes

**Fig. 7:**
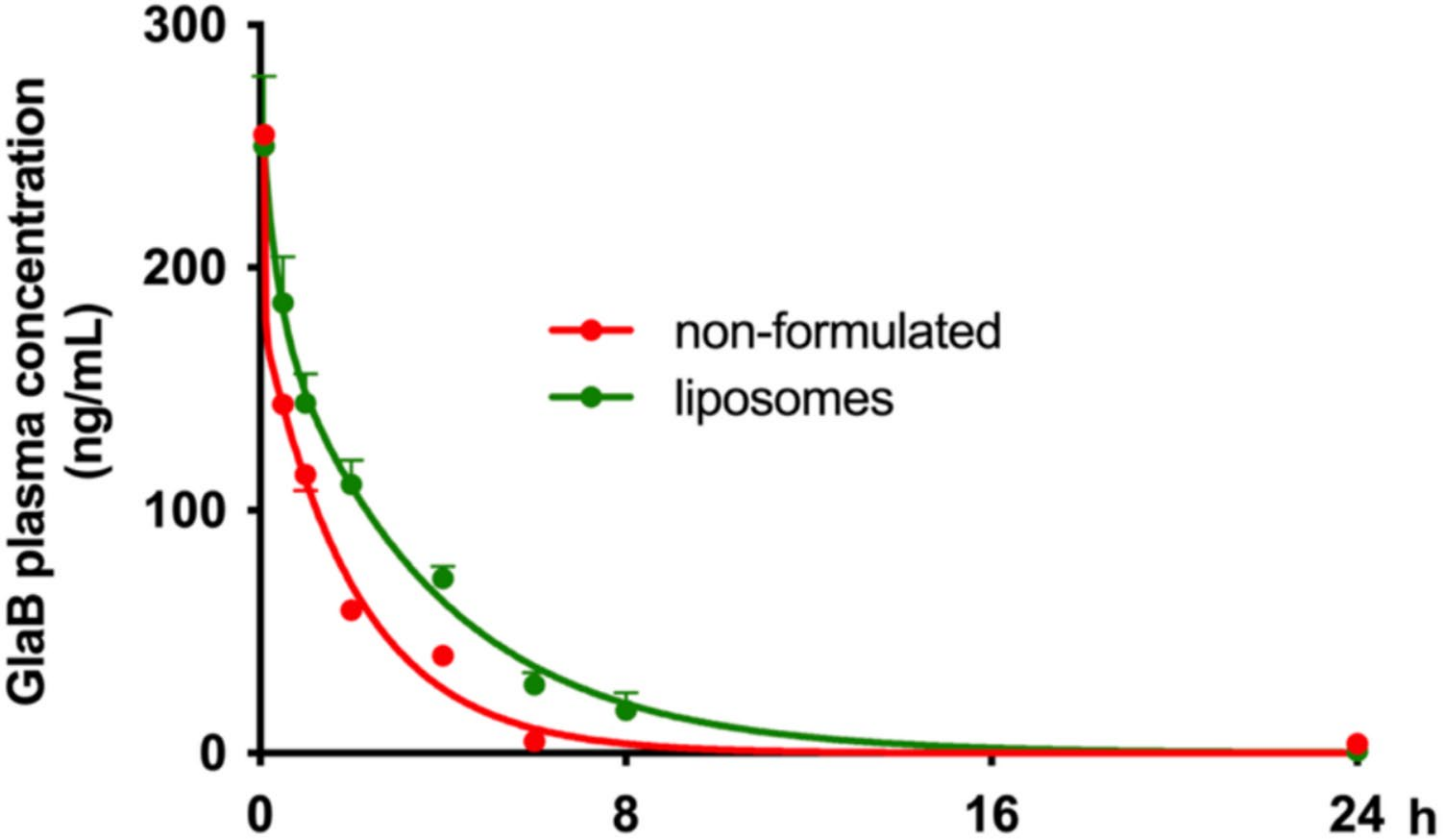
Pharmacokinetic profiles of GlaB-loaded liposomes and non-formulated GlaB in mice. Mean plasma concentration–time profile of GlaB after i.v. administration of a single dose of GlaB (2.5 mg/kg) in the control vehicle (non-formulated) and GlaB-loaded liposomes to c57BL/6 mice (*n *= 4). Data are presented as mean ± SD

The pharmacokinetic (PK) study performed by intravenous (IV) administration of Lipo/GlaB to C57BL/6 mice was aimed at investigating the in vivo behavior of Lipo/GlaB in comparison with previously developed formulations [15] with a view toward future translational studies in orthotopic MB models. The C57BL/6 mouse strain was selected because it is commonly used for PK studies for colloidal formulations of drugs [12]. GlaB dissolved in non-ionic surfactant/DMSO was used as control. This dissolving medium of GlaB used in our studies is a mixture of commonly used components to administer insoluble drugs in preclinical studies [45]. Plasma concentrations of GlaB were quantified by LC–MS using the calibration curve y = 0.00188x + 0.00281 (R^2^ = 0.9993). Although both GlaB-loaded liposomes and control GlaB PK profile fit a biexponential decay, Fig. 7 and Table 1 show that the liposomes modified the GlaB pharmacokinetics.

**Table 1:** Pharmacokinetic parameters of GlaB (mean ± SD)

		Parameters		
		t_1/2α_ (h)	t_1/2β_ (h)	AUC_0-inf_ (ng/mL·h)
Formulation				
GlaB-loaded liposomes		0.265 ± 0.0828**	2.49 ± 0.16**	710 ± 62***
GlaB in surfactant/DMSO		0.0878 ± 0.0347	1.54 ± 0.31	405 ± 26

**p < 0.01, ***p < 0.001, and ****p < 0.0001 vs GlaB in surfactant/DMSO

The α and β half-life constants and the area under the concentration-vs-time curve (AUC_0-inf_) of GlaB formulated in liposomes were 3.0- and 1.6- and 1.75-fold higher, respectively, compared to the values obtained with non-formulated GlaB, indicating the former undergoes to a slower distribution to peripheral tissues and elimination with respect to the latter. The correlation of the GlaB release from liposomes (Fig. 2A) with the α and β half-life constants (Table 1) indicates that the prolonged permanence of GlaB in the bloodstream is mainly attributable to the GlaB retained within the liposomes. Indeed, GlaB was slowly released from liposomes with only 15% released drug in 3 h while the half-life constants are higher than the ones obtained with non-formulated GlaB. The liposome formulation also outperforms one previously published mPEG_5kDa_-cholane/GlaB micellar system [15] that displayed about 15% lower AUC and 35% and 25% lower α and β half-life constants, respectively, than the ones obtained with the Lipo/GlaB. Importantly, long circulating nanoformulations can enhance the tumor exposure and disposition in the Hedgehog (HH)-driven medulloblastoma, which is characterized by relatively impermeable vasculature [46, 47] also in the case of active targeting such as via P-selectin [48]. Therefore, the PK findings are particularly relevant, as they demonstrate that the developed liposomes exhibit promising in vivo profile by IV administration and provide a suitable platform for future targeted delivery strategies in HH-MB.

## Conclusions

In this study we developed a liposomal formulation for GlaB delivery (Lipo/GlaB) with suitable biopharmaceutical properties including high drug encapsulation, high stability in serum and controlled release, which can be exploited for treatment of Hedgehog (HH)-dependent tumors. Lipo/GlaB treatment in pre-clinical models of HH-driven medulloblastoma (MB) and basal cell carcinoma (BCC), which often presents as locally advanced and/or unresectable lesions, was found to inhibit Gli1 transcriptional activity and HH pathway, thus remarkably suppressing the tumor growth. Moreover, the prolonged circulation time upon IV administration suggests that liposomal GlaB formulation possesses the pharmacokinetic requisites for GlaB delivery to the central nervous system tumors such as MB.

Taken together, liposomal formulations, like Lipo/GlaB, represent a drug-delivery platform with high translational potential for clinical applications in the treatment of HH-driven malignancies across different anatomical and pathological contexts.

## Supplementary Information

Below is the link to the electronic supplementary material.

Supplementary file1 (DOCX 190 KB)

## Data Availability

The datasets generated during the current study are available from the corresponding authors on reasonable request.
