# Supplementary material for: Functional and therapeutic effects of Glabrescione B delivery by liposomes on Hedgehog-dependent tumors
Source: Drug Deliv Transl Res. 2025 Dec 16;16(7):2439–55. doi: 10.1007/s13346-025-02026-0 (PMC13294236; doi:10.1007/s13346-025-02026-0)
Supplement: Supplementary file 1 — Supplementary file1 (DOCX 190 KB) [file 13346_2025_2026_MOESM1_ESM.docx]

**Supplementary Information, Infante et al.**

**Table S1.** Primer sequences used for qPCR analysis

| **Gene** | **Forward primer (5'→3')** | **Reverse primer (5'→3')** |
| --- | --- | --- |
| m*Hprt* | GCTTCCTCAGACCGCTT | GGTCATAACCTGGTTCATCATGG |
| m$\beta$*2m* | ACAGTTCCACCCGCCTCACATT | TAGAAAGACCAGTCCTTGCTGAAG |
| m*Gli1* | GCCAACTTTATGTCAGGGTCCCAG | GGAGAGAGCCCGCTTCTTTGTTAA |
| m*Gli2* | CAGCCACCCCAGCGTAGACA | GCCCCAGGTCGCACTCTAG |
| m*CycD1* | TCCGCAAGCATGCACAGA | AGGGTGGGTTGGAAATGAACT |
| m*CycD2* | AGAAGGACATCCAACCGTACATG | CATGGCCAGAGGAAAGACCTC |
| m*Ptc1* | GCATTGGCAGGAGGAGTTGA | AGTCATTAACTGGAACATGGTTTGC |

**Table S2.** Summary of the statistical analysis of liposomes size upon lyophilization in the presence of different lyoprotectants at increasing concentrations and redispersion. Freshly prepared liposomes (BL), liposomes lyophilized without lyoprotectant (NC).

| One-way Anova, Dunnett's multiple comparisons test | **Significant** | **Summary** | **P Value** |
| --- | --- | --- | --- |
|  | | | |
| **BL vs. NC** | Yes | *** | 0,0007 |
|  | | | |
| **BL vs. Mannitol** |  | | |
| BL vs. 1% w/V mannitol | Yes | **** | <0,0001 |
| BL vs. 2% w/V mannitol | Yes | **** | <0,0001 |
| BL vs. 3% w/V mannitol | Yes | * | 0,0490 |
| BL vs. 5% w/V mannitol | No | ns | 0,2297 |
| BL vs. 7% w/V mannitol | No | ns | 0,6166 |
|  |  |  |  |
| **BL vs. Trehalose** |  | | |
| BL vs. 1% w/V trehalose | Yes | **** | <0,0001 |
| BL vs. 2% w/V trehalose | Yes | **** | <0,0001 |
| BL vs. 3% w/V trehalose | No | ns | 0,9967 |
| BL vs. 5% w/V trehalose | No | ns | 0,9999 |
| BL vs. 7% w/V trehalose | No | ns | 0,9998 |
|  |  |  |  |
| **BL vs. Sucrose** |  |  |  |
| BL vs. 1% w/V sucrose | Yes | **** | <0,0001 |
| BL vs. 2% w/V sucrose | Yes | **** | <0,0001 |
| BL vs. 3% w/V sucrose | No | ns | >0,9999 |
| BL vs. 5% w/V sucrose | No | ns | 0,9999 |
| BL vs. 7% w/V sucrose | No | ns | 0,9998 |


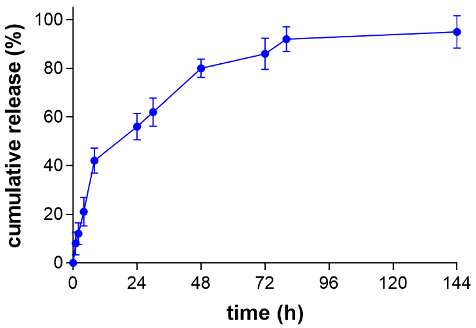


**Figure S1.** GlaB cumulative release from lyophilized and reconstituted liposomes (95:5 mol/mol% EPC/Cholesterol). Release was performed in 10 mM phosphate, 150 mM NaCl, pH 7.4, 37 °C. Mean ± Standard Deviation (SD) of the results were based on four test repetitions.


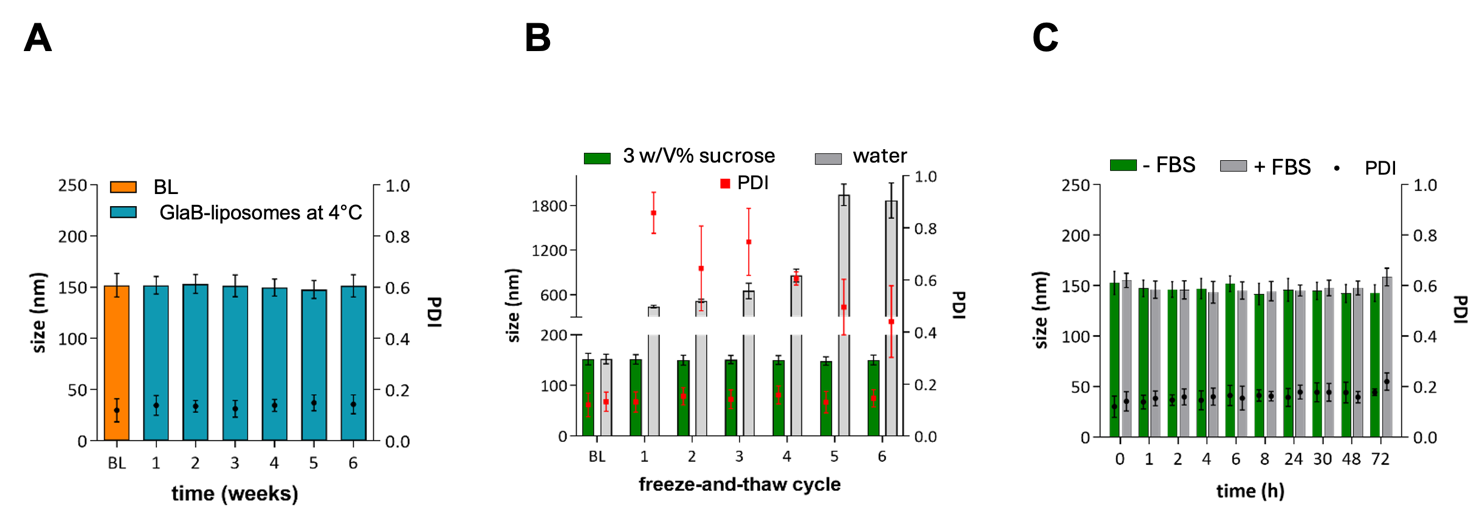


**Figure S2.** (A) Colloidal stability profile of lyophilized GlaB-loaded liposomes (95:5 mol/mol% EPC/Cholesterol) over 6 weeks storage at 4 °C. Liposomes were lyophilized with 3 % w/V sucrose and redispersed immediately before size analysis. Freshly prepared GlaB-loaded liposomes (BL) are reported as reference. (B) Size profile of GlaB-loaded liposomes in water and in water with 3 % w/V sucrose after repeated freeze-and-thaw cycles. Freshly prepared liposomes (BL) are reported as reference. (C) Colloidal stability profile of lyophilized and redispersed GlaB-loaded liposomes in 10 mM phosphate, 150 mM NaCl, pH 7.4 and in 10 mM phosphate, 150 mM NaCl, added of 5 v/v % FBS at 37 °C. Each experiment was independently repeated four times under the same conditions.

**Figure S3.** Cell viability profile of Daoy cells treated with reconstituted GlaB-loaded lyophilized liposomes (95:5 mol/mol% EPC/Cholesterol), free GlaB, reconstituted GlaB-free lyophilized liposomes and sucrose in complete medium. The concentration of GlaB-free lyophilized liposomes and sucrose are equivalent to those of the GlaB-loaded liposomes. Free GlaB was not tested at concentrations of 200-600 μM (≠). Each experiment was independently repeated four times under the same conditions. *p< 0.05; **p < 0.01 vs CTR.
